# Lessons Learned From Shared Decision-Making With Oral Anticoagulants: Viewpoint on Suggestions for the Development of Oral Chemotherapy Decision Aids

**DOI:** 10.2196/56935

**Published:** 2024-09-11

**Authors:** Daniel E McLoughlin, Fabiola M Moreno Echevarria, Sherif M Badawy

**Affiliations:** 1 Northwestern University Feinberg School of Medicine Chicago, IL United States; 2 Division of Hematology, Oncology and Stem Cell Transplant Ann & Robert H. Lurie Children’s Hospital of Chicago Chicago, IL United States

**Keywords:** shared decision-making, SDM, decision aids, decision aids design, oral chemotherapy, oral anticoagulants, drug delivery, chemotherapy, chemo, anticoagulants, drug deliveries, cancer, oncology, oncologist, metastases, literature review, literature reviews

## Abstract

Oral chemotherapy is commonly prescribed, and by using decision aids (DAs), clinicians can facilitate shared decision-making (SDM) to align treatment choices with patient goals and values. Although products exist commercially, little evidence informs the development of DAs targeting the unique challenges of oral chemotherapy. To address this gap in the literature, our objective was to review DAs developed for oral anticoagulation, DA use in oncology, and patient preference surveys to guide the development of DAs for oral chemotherapy. We focused on reviewing SDM, patient preferences, and specifically the development, efficacy, and patient experience of DAs in oral anticoagulation and oncologic conditions, ultimately including conclusions and data from 30 peer-reviewed publications in our viewpoint paper. We found that effective DAs in oral anticoagulation improved knowledge, lowered decisional conflict, increased adherence, and covered a broad range of SDM elements; however, limited information on patient experience was a common shortcoming. In oncology, DAs increased knowledge and aligned decisions with the values of the patients. Ineffective oncology DAs provided general, unclear, or overly optimistic information, while providing “too much” information was not shown to do harm. Patients preferred DAs that included pros and cons, side effects, questions to ask, and expected quality of life changes. In developing DAs for oral chemotherapy, patients should be included in the development process, and DA content should be specifically tailored to patient preferences. Providing DAs ahead of appointments proved more effective than during, and additional considerations included addressing barriers to efficacy. There is a need for evidence-based DAs to facilitate SDM for patients considering oral chemotherapy. Developers should use data from studies in oral anticoagulation, oncology, and preference surveys to optimize SDM.

## Introduction

Oral systemic treatment is becoming an increasingly common modality of anticancer therapy [1]. It may be preferred to traditional intravenous administration due to patient convenience, its noninvasive nature, the safety of prodrugs relative to intravenous “full drugs,” and reducing the costs that accompany additional outpatient appointments and inpatient resource usage [2]. However, it is not without its drawbacks. Adherence can be difficult, absorption can be variable, and administration may be impossible in patients with dysphagia. In addition, administration may be confusing for patients, for example, some drugs (sorafenib) present challenges when taken with food while others (imatinib) must be taken with food [2].

Shared decision-making (SDM), a process through which providers and patients weigh evidence and make decisions together [3] that is becoming increasingly important in clinical practice, can help patients balance these strengths and shortcomings. Studies have shown that patients like playing an active role in care-related decision-making [4,5], clinician education is becoming geared toward facilitating SDM [6], and a lack of SDM has been associated with lower medication adherence, even when concrete decisions are made [7]. To help facilitate SDM, interventions such as mobile apps, videos, or informative visuals, known as decision aids (DAs), can provide patients with information regarding treatment options, their associated risks and benefits, and how drug administration aligns with a patient’s goals and values [8].

In oncology, DAs have been used since at least the advent of the “Decision Board” in 1992 for adjuvant chemotherapy in node-negative breast cancer [9,10], and although considerable research exists on their use and efficacy in oncology as a whole, and products do exist commercially [11], there is little in the current literature examining the development and efficacy of DAs targeting the unique challenges of oral chemotherapy. This article addresses this gap by reviewing what is known about the use of DAs in oncology and using the example of oral anticoagulation DAs to examine how they may be best leveraged to facilitate SDM in oral chemotherapy.

## Decision Aids in Oncology: Patient Preferences, Successes, and Shortcomings

In considering how to approach the development of a DA for oral chemotherapy, we should first consider the preferences of the target population and how DAs have been successfully implemented in the field of oncology. A study involving patients with all tumor types revealed that patients want their DAs to be specific, which may include the pros and cons of each treatment choice and a list of questions to ask their provider [4]. Another study, although only specific to men with prostate cancer, indicated that patients like when DAs include information regarding potential side effects and a clear discussion of the expected quality of life resulting from treatment [12]. These findings could be especially important for patients considering oral chemotherapy, as focusing only on deciding between systemic therapy as a general category versus surgery or observation may miss the unique factors that make a rigid, daily oral medication schedule challenging when compared with hospital-based intravenous treatment. Expected quality of life may be of particular importance to this patient population, as prostate cancer has a more favorable prognosis than many other malignancies. However, it stands to reason that it could be important to patients with less favorable prognoses as well, especially when considering whether they would like such therapy to be a part of the time that they do have left. In addition, patients report that they prefer DAs that are targeted to their specific needs [4], which may extend beyond only their disease process, as there have been calls for DAs in oncology to account for the diversity of patient populations [13].

Regarding how much information to disseminate to patients, a study of men with advanced cancer found that full discussions regarding prognosis decreased depressive symptoms; however, patient anxiety was found to be higher if the clinician felt that such a discussion had taken place [14]. However, it should be noted that this study did not address baseline anxiety or depression before their diagnosis. While this study was specific to men, another study that included men and women found that clinician-driven encouragement to participate in treatment decisions was associated with increased patient anxiety persisting after a 2-week period, suggesting that this change may be independent of baseline anxiety [15]. This raises some concern that providing too much information and involving patients more in decisions regarding their care could be overwhelming or distressing and could ultimately do more harm than good. However, Cripe et al [14] suggest that such results may be due to the content of the provider’s encouragement and discussion: it may not align with patient preferences and therefore contribute to anxiety development. This is supported by the findings of Gattellari et al [15] that information disclosure itself was not associated with increased anxiety. Ultimately, Cripe et al [14] propose that patient anxiety is a signal that further discussions regarding patient goals and preferences should occur. DAs may help facilitate such discussions by including surveys that specifically elicit individual patient values [16], and multigender studies in patients with advanced cancer, including a systematic review, have shown that providing more information through DAs does not do harm [17,18]. Likewise, although it has been observed that patients with advanced gastrointestinal cancer reported lower quality of life scores and higher anxiety if they acknowledged that their illness was terminal [19], DAs can help mitigate this as well, as it has been shown through a meta-analysis of 16 studies of adult patients of varying tumor types that DAs may help reduce anxiety and fear, especially in newly diagnosed patients [16]. Finally, for many patients, additional information may be seen as a positive: 1 study, which interviewed 27 patients with cancer, found that patients nearly always wanted to know as much as possible about cancer as a whole, their prognosis, treatment benefits, and side effects [20].

DAs in oncology have been reported acceptable by both patients and providers [21], have been shown to increase patient knowledge [9,16], and lower decisional conflict, aligning patients’ ultimate decisions with their personal values [16,22]. In addition, DAs in cancer care have been shown to increase patient satisfaction with both the information presented and their treatment decision [4,9,16]. However, it is worth acknowledging that the mere presence of a DA is insufficient. A study of a DA for oral complementary and alternative medications for patients on chemotherapy, which provided predominantly general information, did not help decrease decisional conflict or patient regret [23]. DAs presenting unclear or overly optimistic information, especially regarding side effects, have been shown to provide patients with a worse experience, as this can lead to a misperception of the risks and benefits of treatment and may ultimately affect decision-making [17].

## Oral Anticoagulation Decision Aids as a Model

With similar treatment schedules, required monitoring, and experience of self-administering medication, oral anticoagulation presents similar advantages and challenges to oral chemotherapy. As significant data exists regarding the efficacy and implementation of DAs in oral anticoagulation, these findings can be used to help inform the development of evidence-based DAs in oral chemotherapy. Using DAs in oral anticoagulation has been shown to help with improving patient knowledge [21,24,25], lowering decisional conflict [21,24-27], increasing medication adherence [24], and increasing the likelihood of making a choice [25].

Oral anticoagulation DAs considered effective, defined as improving health outcomes or at least increasing or enhancing SDM, have focused on covering a broad range of 6 SDM elements, that are situation diagnosis, choice awareness, option clarification, discussion of harms and benefits, deliberation of patient preferences, and decision-making [24]. In a study of 10 DAs in which 7 were deemed successful, 6 included discussions of harms and benefits and at least one of choice awareness and deliberation of patient preferences. This suggests that, although all merit inclusion, these 3 may be the most critical to consider when developing DAs for patients contemplating oral therapy [24].

Oral anticoagulation DA studies can also demonstrate what has not been successful in DA development. Logically, including a narrower range of the 6 elements of SDM does not support efficacy [24]. In addition, a review of 14 SDM tools focused on choosing between Warfarin and direct oral anticoagulants from Torres Roldan et al [28] showed that the current DA developmental process rarely includes patients. The studies observed were overall unsuccessful, as only 2 of the 14 DAs reviewed improved adherence, and 3 of the 14 did not support SDM. The authors note that a common shortcoming of these DAs was that they lack information on the day-to-day patient experience, including “what it means to take a pill every day” and “what it takes to attend periodic clinic appointments” [28]. This demonstrates that, despite the fact that they usually did include good information regarding treatment options, outcomes, prognosis, costs, dosing, and side effects, these DAs may have fallen short of their potential maximum effect [28]. Involving the patient in the developmental process could help fine-tune DA content to include information that will most benefit patients.

Although it should be noted that the disease processes themselves (hypercoagulable state vs malignancy) carry significantly different clinical implications, which could influence patient priorities when using DAs, the advantages and challenges of the administration of oral anticoagulation and oral chemotherapy are similar. Therefore, this information should be used in conjunction with what is known about DA use in oncology to develop DAs ideally suited for patients with cancer contemplating oral therapy.

## The Ideal Design of an Oral Chemotherapy Decision Aid

### Patient Involvement in Decision Aid Development

**Figure 1:**
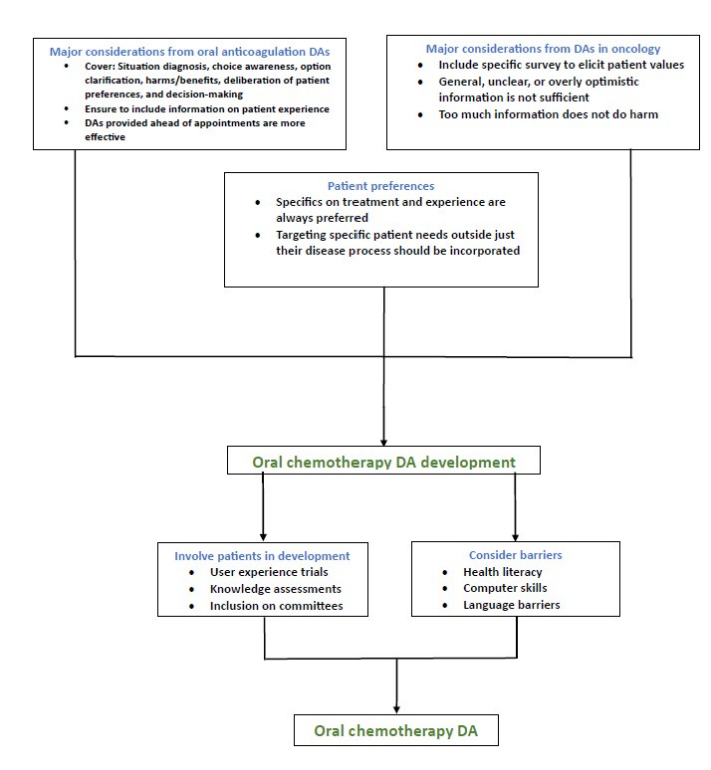
Schematic of major considerations in the development of decision aids for oral chemotherapy, demonstrating the use of knowledge gained from decision aids in oral anticoagulation, oncology, and patient preference surveys in the predevelopment process and the involvement of patients and consideration of barriers in the development process. DAs: decision aids.

Data regarding patient preferences, current DA use in oncology, and DAs in oral anticoagulation serve as a framework for informing what the development, implementation, and DA product itself should look like for oral chemotherapy (Figure 1). Most importantly, patients should be involved in the developmental process from its early stages. Doing so would help direct focus toward user experience, and it also allows for early identification of issues and provides time for modifications. For example, a common shortcoming of DAs in both oncology and oral anticoagulation is that they can lead patients to have an inadequate perception of risk [17,26]. It has been shown that involving patients early can help minimize this; for example, 1 DA for oral anticoagulation, which did have patients involved during development, identified this issue early in the process, and developers were able to adjust by incorporating a user-friendly, color-coded visual depiction of risk level in the next version of the application [26]. In addition, although DAs often improve patient knowledge [9,16,21,24,25], 1 study on DAs for second-line palliative chemotherapy demonstrated improved subjective knowledge, which is the patient’s perception of their own understanding, but not objective knowledge [17]. We propose that the difference between the 2 could be teased apart by running pilot tests, including knowledge assessments, with patients during the developmental process. These assessments should be geared toward answering the question “Does this convey the information necessary for a patient to make an informed decision?” If not, modifications can be made.

### Tailoring Decision Aids to Patient Needs

Regarding the dissemination of information, DAs should be targeted to specific patient needs and include specific information, including regarding side effects, which has been shown to be important for patients with cancer [4,12]. Surveys incorporated into a DA’s interface that elicit user values can help, especially in selecting a treatment that best aligns with their values. However, the challenge of integrating a simple, user-friendly interface with sufficient detail to provide patients with the information necessary to make complex medical decisions remains. A small pilot study of just 27 patients of varying genders, educational backgrounds, and tumor types did find that nearly all of their patients preferred to know as much as possible [20], but health literacy may vary, and different patients may require or prefer different levels of nuanced discussion. An example of a successful DA for oral anticoagulation included a multi-tiered system in which the main points were presented on one page, and additional links were included that provided options for patients to receive more granular, detailed information if they chose [26]. This should serve as a framework for the development of any DA for similar treatment modalities, including oral chemotherapy, as it can help disseminate an appropriate level of information to a large range of patients. For example, it would be essential for all patients to understand “what it means to take a pill every day,” as described by Torres Roldan et al [28], and this, along with basic side effect information, could be included on the main page. Meanwhile, the “linked” pages could have detailed example schedules for both dosing and follow-up appointments, explanations of drug mechanisms, and detailed side-effect profiles.

### Inclusion of Rarer Diseases

Tailoring DAs to the specific needs of patients [4] should include developing aids for less common indications and treatment options. A systematic review on DAs for SDM in urologic malignancies found 22 DAs available for prostate cancer and just 2 for renal cancer and 1 for bladder cancer [29], demonstrating a need for DAs in rarer diseases. Another systematic review that examined DA use in decisions that include “active surveillance” as a management option found that, despite active surveillance being used in colorectal, thyroid, and head and neck cancer management, 21 of the 23 included studies were focused specifically on prostate cancer [30], which also suggests a need for further investigation of DA use in a wider spectrum of malignancies. In developing DAs for oral chemotherapy, consideration should be given to rarer indications and diseases. Incorporating additional links within the interface of an app directed at a specific therapy could be one method of including information that may be critically relevant to a subset of patients with a particular disease or comorbidity. It bears mentioning that such considerations, while mentioned in this context specifically for oral chemotherapy, may also benefit patients in oncology as a whole, as options for patients with rarer diseases often extend beyond only the nuances of oral treatment and may require further education that has thus far been marginalized in DA development.

### Addressing Barriers to Success in the Clinical Setting

Developing successful DAs for oral chemotherapy also requires considering potential barriers to efficacy. If not considered during development, language and computer literacy can present challenges, so alternative methods of delivering information [16] within the same interface may be necessary because the information is useless if it is not accessible. Incorporation of an audio option within an app could be an example, and although additional studies would be needed to examine the relative efficacy of audio versus textual dissemination of information, this is yet another reason to involve patients in the development and testing processes. In addition, although it has been shown that DAs can be effective in populations of lower socioeconomic status [21,26], this also requires targeted delivery, as patients may have limited funds for an application or downloadable content. One proposition to address this includes preloading health-related content on mobile devices analogous to how many cell phones are preloaded with games; alternatively, DAs could be presented as open-access downloadable content [31]. Similar to using DAs for rarer diseases, it should be noted that addressing barriers to access and optimal information delivery is not specific to oral chemotherapy and can have wide-reaching implications in DA development in oncology and beyond.

### Timing of Decision Aid Delivery

Essential consideration should also be given to the timing of DA presentation to patients. Current literature shows that doing so before a treatment consultation or discussion provides an evidence-based method of maximizing efficacy. Again, using oral anticoagulation as a model, the study by Song et al [24] of 10 DAs that found 7 to be considered “effective” demonstrated that all 3 ineffective DAs were given to patients during a consult, while 5 of the 7 effective ones were provided in advance. In other words, all 5 DAs provided ahead of a consult were effective, while only 2 of the 5 provided during the consult were. The authors postulate that this is because patients have time to digest information ahead of their appointment [24], and another potential factor may be that doing so allows for time to formulate clarifying questions [16], especially since patients have indicated that they like when DAs include a list of questions worth asking their provider [4]. Literature also shows that, for patients with cancer, the use of DAs can also help increase caregiver involvement [16]. Providing DAs ahead of a consult would increase opportunities for patients to discuss their thoughts, preferences, and concerns with caregivers and family members if they choose. Importantly, consideration should be given to the possibility of increased anxiety that may occur if patients are encouraged to participate in their care before the eliciting of patient preferences [14]. It would likely be important to include an accompanying note that briefly describes the type of information included in the DA and an explanation that its contents could be reviewed at the appointment if the patient would prefer to go over it with a clinician first. This would provide patients with the opportunity to review in advance and maximize potential efficacy, but it would also provide a safeguard of an initial review with their provider if they would prefer.

## Further Investigation

Once the initial developmental process is complete, trialing the DA could commence. User experience trials evaluating DA design, experience with the interface, and perceptions of ease of use would likely occur first and would be best optimized with user response surveys. To optimize feedback and maximize the impact of patient perspectives, qualitative and quantitative data should be collected. Panels that include user experience specialists, product designers, physicians, and volunteer patients could then meet on developmental committees to fine-tune the pilot DA based on this feedback. Although practical considerations would likely limit patient selection at this stage to a convenience sample, it would provide valuable insight into the patient experience of using the DA before optimizing its clinical use.

This would be followed by knowledge assessments conducted through randomized controlled trials in a simulated environment. Knowledge retention would be compared between individuals provided information through standardized clinical encounters with a physician versus those who were also provided with the DA. If this trial demonstrates efficacy (improved SDM), the product then can move to clinical practice in select environments, with ongoing quality improvement studies to optimize their use. These would ideally be set up as prospective cohort studies but could also use a case-control or retrospective cohort design if institution-specific questions regarding their use and implementation arise.

In addition, many questions remain unanswered, warranting further critical discussion and investigation. Providing DAs ahead of appointments may be beneficial relative to during appointments [24], but is there a need for further outreach to maximize the uptake rate and limit potential patient anxiety? Although difficult to concretely define the specific needs of individual patients [4], would involving technological user experience specialists in development help get us closer to doing so? As Bennett et al [13] allude to, how can we leverage DAs to address inherent shortcomings in communication, especially bias, from the clinician side?

## Limitations

As this paper is presented as a viewpoint, a review of relevant literature was conducted in a nonsystematic manner. This may subject the paper to reviewer bias and does render it possible that potentially pertinent articles were not included. However, the purpose of this viewpoint and the associated literature review is not to provide a definitive, comprehensive state of multiple fields, as there is no extant literature on the development and implementation of DAs for oral chemotherapy. Rather, its purpose is to take the initial steps to address this gap in the literature by using evidence from related fields (DA use in oncology and oral anticoagulation) to provide suggestions for the development of oral chemotherapy–specific DAs. Ultimately, its aim is to inform future research so that evidence-based guidelines may be developed in the future.

## Conclusions

There is a need for evidence-based, effective DAs to facilitate SDM for patients considering oral chemotherapy. Important considerations in the development of these DAs include including a broad range of SDM elements, involving patients in the development process, tailoring content to specific patient needs, and anticipating and addressing potential barriers to efficacy. Further research is needed to investigate the efficacy of DAs developed specifically for oral chemotherapy.

## Abbreviations

DA: decision aid
SDM: shared decision-making
